# Highly Stretchable Composite Conductive Fibers (SCCFs) and Their Applications

**DOI:** 10.3390/polym16192710

**Published:** 2024-09-25

**Authors:** Diane Tang, Ruixiang Qu, Huacui Xiang, Enjian He, Hanshi Hu, Zhijun Ma, Guojun Liu, Yen Wei, Jiujiang Ji

**Affiliations:** 1 The Key Laboratory of Bioorganic Phosphorus Chemistry & Chemical Biology, Department of Chemistry, Tsinghua University, Beijing 100084, China; diane.tang@some.ox.ac.uk (D.T.); huac_xiang@163.com (H.X.); hej21@mails.tsinghua.edu.cn (E.H.); hshu@mail.tsinghua.edu.cn (H.H.); 2 Inorganic Chemistry Laboratory, University of Oxford, South Parks Road, Oxford OX1 3QR, UK; 3 Zhejiang Lab, Hangzhou 310000, China; qrx21@zhejianglab.edu.cn (R.Q.); zhijma@zhejianglab.com (Z.M.); 4 Department of Chemistry, Queen’s University, 90 Bader Lane, Kingston, ON K7L 3N6, Canada; 5 Department of Chemistry, Center for Nanotechnology and Institute of Biomedical Technology, Chung-Yuan Christian University, Taoyuan 32023, Taiwan

**Keywords:** stretchable composite conductive fibers, conductive filler, wearable electronics, bioelectronics

## Abstract

Stretchable composite conductive fibers (SCCFs) exhibit remarkable conductivity, stretchability, breathability, and biocompatibility, making them ideal candidates for wearable electronics and bioelectronics. The exploitation of SCCFs in electronic devices requires a careful balance of many aspects, including material selection and process methodologies, to address the complex challenges associated with their electrical and mechanical properties. In this review, we elucidate the conductive mechanism of SCCFs and summarize strategies for integrating various conductors with stretchable fibers, emphasizing the primary challenges in fabricating highly conductive fibers. Furthermore, we explore the multifaceted applications of SCCFs-based frameworks in wearable electronic devices. This review aims to emphasize the significance of SCCFs and offers insights into their conductive mechanisms, material selection, manufacturing technologies, and performance improvement. Hopefully, it can guide the innovative development of SCCFs and broaden their application potential.

## 1. Introduction

The miniaturization and integration of electronic devices have significantly enhanced the portability of wearable electronics, including smartwatches, sports bands, electronic skins, etc [1,2,3,4]. In general, wearable systems, composed of carrier materials, power supplies, sensors, and communication modules, can monitor physiological parameters such as temperature, blood pressure, and various ions and biomolecules in blood [5,6,7]. These systems are increasingly vital in healthcare, virtual and augmented reality, health monitoring, and human–computer interaction [8,9]. Thin-film structures have dominated wearable electronics in the past decades, enabling the integration of electronic devices and systems on flexible thin-film substrates. However, epidermal thin-film devices often cause occlusion, leading to discomfort and irritation with prolonged use [10,11,12]. Ideal bioelectronics should protect the skin or tissues from infection, irritation, inflammation, or immune response and provide a safe environment for users. Recently, fiber-based frameworks have emerged as a promising alternative. These fibers and fiber assemblies, as a unique class of soft materials, exhibit lightweight characteristics, remarkable flexibility, excellent permeability, and structural diversity [13,14]. Therefore, fiber-based conductive materials are among the most promising materials for wearable electronic devices, given their small footprint and exceptional stretchability, conductivity, and permeability [14,15,16]. With appropriate conductive fiber composition and the application of mature weaving, knitting, and structural design techniques, it becomes feasible to meet the requirements for both ideal wearable devices and biological interface electronics [17,18].

Textile-based electronics constitute a highly promising facet of wearable technology. At the intersection of stretchable electronics and fibers, stretchable composite conductive fibers (SCCFs) have rapidly emerged as a material of great interest [19,20]. SCCFs can be fabricated either by directly processing intrinsically conductive stretchable materials into fibers or by applying post-treatments to impart conductivity to stretchable fibers [21,22,23]. These systems rely on conductive fibers or yarns to facilitate electrical conduction, signal transmission, and information exchange. The integration of the advantages of stretchable electronics and fibers positions SCCFs as a burgeoning research focus [4,24,25]. Despite their many advantages over traditional conductive materials like rigid wires and conductive resins, SCCFs face the critical obstacle of balancing stretchability and conductivity within a confined cross-sectional area [15,26]. Stretching the fibers can disrupt their conductive pathway, leading to decreased conductivity and compromised device performance. The optimization of stretchability and conductivity in SCCFs requires a thorough understanding of their composition, structure, and practical applications [27,28]. Although extensive research has been conducted on SCCFs, unresolved scientific challenges have hindered their commercialization. Thus, it is crucial to summarize the conductive materials, preparation methods, and applications of SCCFs; explore the intrinsic relationship between their structure and application; and enhance their performance in practical applications [29,30,31].

Herein, we systematically review the research progress on SCCFs from three key aspects: conductive mechanism, conductive fillers, and wearable applications. Firstly, we introduce the conductive mechanism of elastic fibers. Secondly, we explore strategies for integrating commonly used conductors and stretchable fibers, emphasizing the primary challenges in fabricating highly conductive fibers and proposing potential solutions. Finally, we explore the diverse applications of SCCFs-based integrated devices in wearable and biological interfaces, including energy sensors, electronic skin, wound healing, biosensing, etc. Through a comprehensive introduction and illustration of SCCFs, this article aims to provide valuable guidance for future research, advancing the field to new heights.

### 1.1. Materials and Characteristics of SCCFs

Stretchable electronics exhibit significant potential for applications in wearable electronics, soft robotics, medical implants, and biological actuators. As essential components for stretchable electronics, stretchable conductors have been extensively studied to optimize their material and structural properties [32,33]. Over the past few decades, most stretchable electronics have been designed using thin-film structures, e.g., electronic devices and systems are built on elastic thin-film substrates. In contrast, fiber-based stretchable electronics provide enhanced conformity to the body’s contours; greater resilience to deformations during usage; and superior breathability for air, moisture, and body fluids [34,35]. Conductive fibers have evolved through three generations: (I) The first generation consists of flexible conductive fibers made from traditional metals, designed with stretchable structures. However, these fibers face challenges such as poor wearability and conductive instability. (II) The second generation employs polymers as elastic conductive materials, offering advantages such as simple, customizable preparation processes and abundant material sources, although their stability and conductivity remain suboptimal. (III) The third generation utilizes specialized textile yarns to produce composite elastic conductive fibers [28,36]. Conductive fibers, generally defined as fibers with resistivity below 10^7^ Ω cm (200 °C, 65% Relative Humidity), can be categorized into two main types: intrinsic conductive fibers and composite conductive fibers [37,38]. Intrinsic conductive fibers possess inherent electrical conductivity due to their electron transport mechanisms, involving either free or delocalized electrons. Examples include metal fibers [39,40], carbon fibers [41,42], conductive polymer fibers [43,44], carbon nanotube (CNT) fibers [45,46], graphene fibers [47,48], and so on. These fibers demonstrate excellent conductivity, high mechanical strength, and environmental stability, making them ideal for diverse applications. These conductive fibers have great potential for various wearable applications. Despite their advantages, intrinsic conductive fibers often exhibit relatively poor deformation and tensile properties [49,50]. Twisting or coiling conductive fibers is an effective strategy to improve elasticity. Carbon-based fibers with hierarchically twisted/coiled structures show enhanced stretchability, making them suitable for applications such as elastic electrodes, sensors, actuators, artificial muscles, energy transducers, and energy-storage devices [51,52,53].

**Figure 1:**
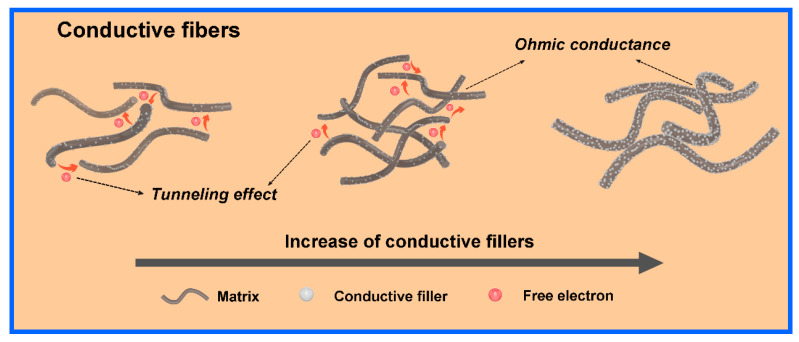
Schematic illustration of the conductive mechanism of conductive fibers.

Stretchable composite conductive fibers (SCCFs) are essential components for fabricating textile electronics, offering high stretchability and conductivity, and serving as electrodes for current collection and signal transmission [14]. SCCFs are composite fibers that integrate multiple functional components to achieve both conductivity and stretchability [27,54,55]. These fibers contain conductive fillers that provide electrical conductivity and insulating polymeric networks that offer structural support for the fillers. Since conventional polymers and fibers are typically insulators, micro-/nanofillers are added to create a conductive network that percolates through the material. This network can either be reinforced by existing elastic fibers or incorporated within elastomeric matrices to fabricate stretchable conductive fibers [56,57]. Percolation, tunneling, and field emission are the three main electrical conduction mechanisms of composite conductive materials [58]. Percolation theory suggests that the conductivity increases abruptly once the volume fraction of conductive fillers reaches a critical threshold (percolation threshold, $p_{c}$). At this point, a conductive network forms through the interconnection of fillers, enabling electron migration and the resulting electrical conduction. Based on Flory’s gelation theory, Kirkpatrick [58] developed a classical statistical percolation model, represented by Equation (1), which describes the relationship between filler loading above $p_{c}$ and the conductivity of stretchable conductive fibers:

(1) $$\sigma =\sigma_{0}{\left(p-p_{c}\right)}^{t}$$

where p is the volume fraction of the conductive filler; σ and $\sigma_{0}$ are the conductivities of the stretchable conductive fibers and the conductive filler, respectively; and t is a scaling factor reflecting the dimensionality of the conductive network within the stretchable conductive fibers. Typically, two-dimensional (2D) conductive networks form when *t* ranges from 1.1 to 1.3, while three-dimensional (3D) networks emerge when *t* falls between 1.6 and 2.0 [59]. As shown in Figure 1, the tunneling effect theory explains electron transfer across gaps between conductive fillers in composite materials under thermal fluctuations. The tunneling effect is predominant in stretchable conductive fibers with low filler loading, where most fillers are encapsulated by a polymer layer during mixing, creating a potential barrier higher than the free electron energy. In classical mechanics, free electrons with energy below the barrier cannot cross it, hindering electronic conduction. However, quantum mechanics shows that microscopic particles with energies below the barrier can still pass through it with finite probability, a phenomenon known as the tunneling effect. Balberg [60] proposed a tunneling conduction model expressed by Equation (2):

(2) $$\sigma_{tun}\propto \exp\left(-r/d\right)$$

where $\sigma_{tun}$ is the tunneling conductivity, r is the tunneling distance, and d is the tunneling decay parameter. The inverse proportionality between $\sigma_{tun}$ and r highlights r as a critical factor in charge transfer between neighboring conductive fillers. Once the filler loading reaches the percolation threshold, conductive networks form within the polymer matrix, offering low-resistance paths for electron transport. Thus, Ohmic conduction becomes the dominant mechanism in this situation. In addition, based on the field emission theory, a high voltage applied to sparsely distributed conductive fillers can generate a strong internal electric field, which allows electrons to overcome the barrier created by the polymer matrices and reach neighboring conductive fillers, resulting in a field emission current.

Stretchability means that the fiber can withstand several deformations, such as bending, twisting, and stretching. This property is typically achieved by either using elastic fibers to reinforce conductive fillers or incorporating an elastic matrix to safeguard the fillers and assemble the fibers. Elastic substrates, such as thermoplastic polyurethane (TPU); styrene–butadiene–styrene (SBS); styrene–ethylene–butylene–styrene (SEBS); and rubber, ecoflex, and poly (dimethylsiloxane) (PDMS), are widely used polymers characterized by a large elongation ability and flexibility. These properties make them suitable for fabricating strain sensors across a broad strain range [61,62,63]. Meanwhile, based on their elemental compositions and conductive mechanisms, commonly used conductive materials for stretchable conductive fibers can be classified into four main groups: metal fillers, carbon-based fillers, conductive polymers, and ionic conductors [58,64].

### 1.2. Carbon-Based SCCFs

**Figure 2:**
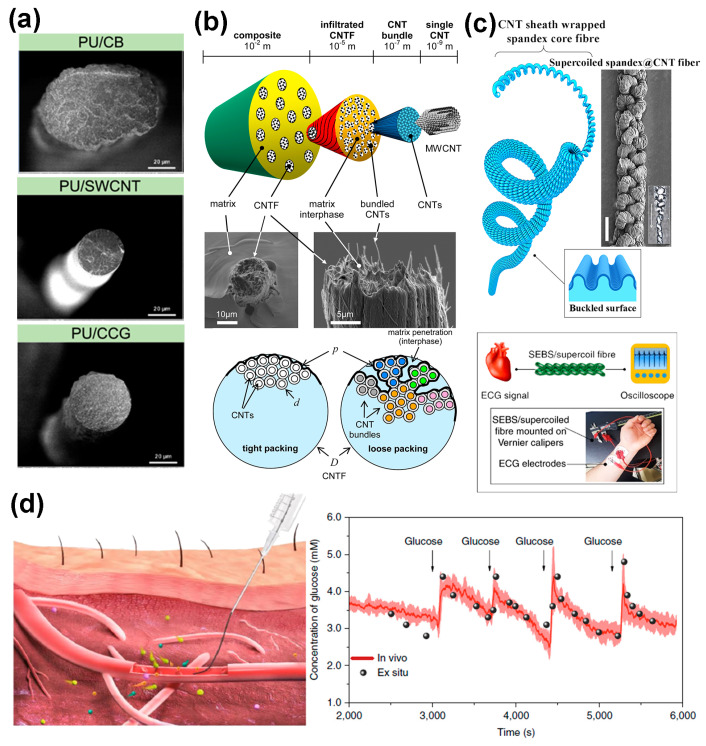
(**a**) SEM images of wet-spun composite fibers: PU/CB, PU/SWCNT, and PU/CCG [68]. (**b**) Composite structure containing CNTs, where each fiber is composed of multiple bundles, with each bundle consisting of thousands of individual CNTs [69]. (**c**) Highly twisted CNT fiber with first-coils and supercoils, along with their applications [70]. (**d**) Injection process of the fiber sensor into a blood vessel for monitoring biological signals [71].

Carbon-based fillers have been demonstrated to be a type of conductive fillers with comprehensive advantages, including superior machinability, mechanical strength, corrosion resistance, and environmental stability [65]. The strain-sensing behaviors of stretchable conductive fibers filled with different dimensional carbonaceous fillers—0D carbon black (CB), 1D carbon nanotubes (CNTs), and 2D graphene—have been widely researched and compared, showing great potential for developing carbon-based strain sensors with desirable properties [66,67]. A key challenge in achieving electrical connectivity with 0D materials is their need for a higher aggregation state, which complicates the trade-off between filler consumption and electrical conductivity. Seyedin et al. [68] investigated how the aspect ratio and interfacial interactions of conductive fillers affect the electrical and mechanical properties of composite fibers. Their findings indicated that fibers with single-wall CNT (SWCNT) fillers exhibited the highest electrical conductivity and mechanical enhancement, while fibers with CB showed the greatest tensile strength. This was because 1D and 2D fillers have fewer resistive junctions per unit area and thus lower percolation thresholds than 0D fillers (Figure 2a). Moreover, composite materials exhibit tendon-like structural hierarchies that significantly enhance strength and toughness. For instance, Sui et al. [69] developed a multilevel composite structure by embedding CNTs into epoxy matrices, resulting in a composite fiber with a hierarchical internal structure (Figure 2b). This structure included four levels of organization: individual CNTs, CNT bundles (where the matrix does not infiltrate), CNT fibers (CNTFs, with or without matrix infiltration), and the composite (CNTFs embedded in the matrix). They employed a modified Cottrell–Kelly–Tyson model to assess matrix permeability effects on fiber–matrix interface properties, demonstrating that the composite’s strength and toughness are closely related to permeability. Direct blending alone does not significantly enhance fiber tensile properties; however, structural design improvements can. Wonkyeong et al. [70] fabricated highly twisted supercoil fibers by inserting a giant twist into spandex-core fibers wrapped in a CNT sheath (Figure 2c). This structure significantly enhanced its stretchability, withstanding up to 1500% elastic deformation. When coated with a passivation layer, the supercoiled fiber showed only a 4.2% increase in resistance when stretched by 1000%. Moreover, this fiber could function as a cable or transmission line, capable of extending up to 11 times its initial length with minimal resistance change. These properties make it suitable for applications in the transmission of physiological signals such as electrocardiogram (ECG) and electromyographic (EMG) signals. Carbon-based stretchable fibers exhibit distinct advantages as implantable sensors due to their minimal biological harm and stable signal transmission. Wang et al. [71] designed a flexible fiber-based implantable sensor that replicates the hierarchical and helical structure of natural soft tissues (Figure 2d). This CNT fiber biosensor, developed by twisting CNTs into bundles, exhibited excellent monitoring performance and could successfully capture electrochemical signals from cats using current-mode (glucose sensor) and voltage-mode (ion sensor) devices.

Overall, carbon-based conductive fillers have attracted considerable attention in the field of stretchable conductive fibers due to their availability, low cost, and ease of fabrication. However, challenges such as poor dispersion, unstable conductive networks, and difficulties in mass production restrict their wider use as conductive components of stretchable electronics. Future efforts should therefore prioritize the development of economical processing methods and advanced modification strategies for carbon-based materials to improve their processability and practical applicability in stretchable conductive fibers [14,58,66].

### 1.3. Metal-Based SCCFs

**Figure 3:**
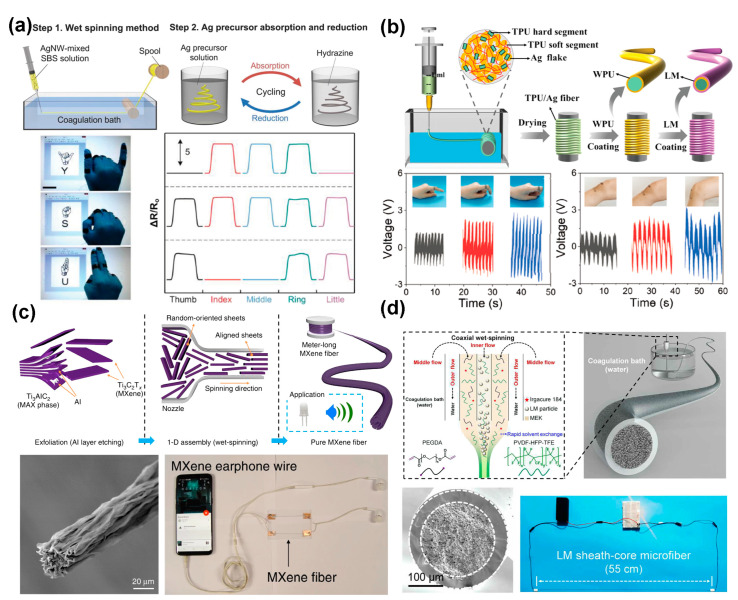
(**a**) Schematic of fabrication and application of the highly stretchable AgNWs/AgNPs/SBS conductive fiber [21]. (**b**) Preparation of TAWL fiber and its application as a stress sensor [77]. (**c**) Wet-spinning process of MXene fiber, including SEM images and applications for transmitting electrical signal to earphones [70]. (**d**) Coaxial wet-spinning process for producing conductive sheath-core microfibers, with cross-sectional SEM of the microfiber and its potential applications [88].

Metals are the most common conductive materials in human daily life, known for their high mechanical strength, electrical conductivity (5 × 10^5^ S·cm^−1^), and thermal conductivity [72]. Compared to carbon-based fillers, metallic materials offer greater potential as conductive fillers because of their various availability, superior electrical conductivities, and mechanical robustness. Among traditional metal conductive materials, Ag possesses the highest conductivity at 6.3 × 10^7^ S·cm^−1^. Silver-related conductive fillers, such as 0D silver nanoparticles (AgNPs), 1D silver nanowires (AgNWs), and 2D Ag flakes (AgFKs), have been widely employed in the fabrication of stretchable conductive fibers [73,74,75]. Lee et al. [21] developed a highly stretchable conductive fiber by embedding AgNWs and AgNPs into an SBS elastomeric matrix (Figure 3a). The embedded AgNWs act as conductive bridges between AgNPs during stretching, allowing the fiber to maintain electrical conductivity under high-strain conditions (with a conductivity retention ratio (*σ*/*σ*_0_) of 4.4% at 100% strain). This conductive composite fiber was made into a strain sensor to monitor the bending motion of a finger joint. As the fiber bent, the resistance of the strain gauge sensor increased rapidly, demonstrating a strong correlation between finger bending and resistance changes. While AgFKs have been used to fabricate stretchable conductive electrodes, their application is less widespread compared to other silver-based materials. AgFKs have high aspect ratios, which confer flexibility and stretchability to the conductive structure [76]. As illustrated in Figure 3b, Zhang et al. [77] utilized a wet-spinning method to create TPU/AgFKs composite fibers, which were then coated with a thin layer of water-borne polyurethane (WPU) to enhance the adhesion between the fiber surface and a subsequent liquid metal (LM) coating. This composite fiber exhibited exceptional mechanical and electrical properties, including a high elongation of ~600% strain, an electrical conductivity of ~2 Ω·cm^−1^ (~3125 S·cm^−1^), and a reversible resistance response under a tensile strain range of 70%. This conductive fiber was used as a wearable, self-powered sensor for detecting human motion. By leveraging the triboelectric effect between the fiber-based sensor and human skin, the sensor generated electricity signals in response to interaction force and device deformation, enabling the detection of various movements.

Besides conventional metal conductors, 2D transition metal carbides and nitrides (MXenes) have attracted considerable attention due to their superior properties, including excellent electrochemical conductivity, a wide variety of terminal groups, layered structures with a large surface area, and hydrophilicity [78,79,80,81,82,83]. As shown in Figure 3c, Eom et al. [84] developed a simple and scalable wet-spinning assembly method to fabricate pure MXene fibers without any additives or binders. These MXene fibers had an electrical conductivity of 7713 S·cm^−1^, which was about 107 and 27 times higher than that of MXene/graphene hybrid fibers (72.3 and 290 S·cm^−1^, respectively) [9] and five times higher than that of MXene/PEDOT:PSS fibers (1490 S·cm^−1^) [85], as previously reported. Moreover, these Ti_3_C_2_T_x_ MXene fibers were used as electrical wires to demonstrate the switching on of an LED light and the transmission of electrical signals to earphones, showcasing their potential for miniaturized portable devices. In addition, liquid metal, specifically eutectic gallium–indium (EGaIn), has emerged as a promising material for fabricating metal-based conductive fibers due to its low melting point (29.8 °C), low viscosity, high surface tension, high electrical conductivity (3.4 × 10^4^ S·cm^−1^), and good thermal conductivity [86,87]. As shown in Figure 3d, Zheng et al. [88] designed a triaxial wet-spinning needle with inner, middle, and outer diameters of 400, 1950, and 3000 μm, respectively. The core of these fibers contained embedded conductive particles, which conformally deformed under stretching due to the sheath–core structure and the dipole–dipole interactions between the fluoroelastomer and the EGaIn. This fiber exhibited impressive mechanical and electrical performance, including large-scale fabrication capability, a maximum strain of ~1170%, a resistance change of only 4% at 200% strain, a high initial conductivity of ~4.35 × 10^4^ S·m^−1^, and a relatively low modulus of ~2.16 MPa. The excellent performance of this fiber in electrothermal discoloration and self-powered sensing positions it as a promising candidate for applications in smart textiles and wearable sensors.

Metallic conductive fillers are the most common conductive elements for fabricating SCCFs. However, certain drawbacks, such as a high Young’s modulus, low compliance, and poor chemical stability, need to be carefully considered during fabrication. Deformable LMs have attracted increasing attention due to their outstanding physical properties, including a low melting point, excellent fluidity, high electrical conductivity, and low toxicity, making them highly promising for use in stretchable electronic devices [89,90].

### 1.4. Conductive Polymer-Based SCCFs

**Figure 4:**
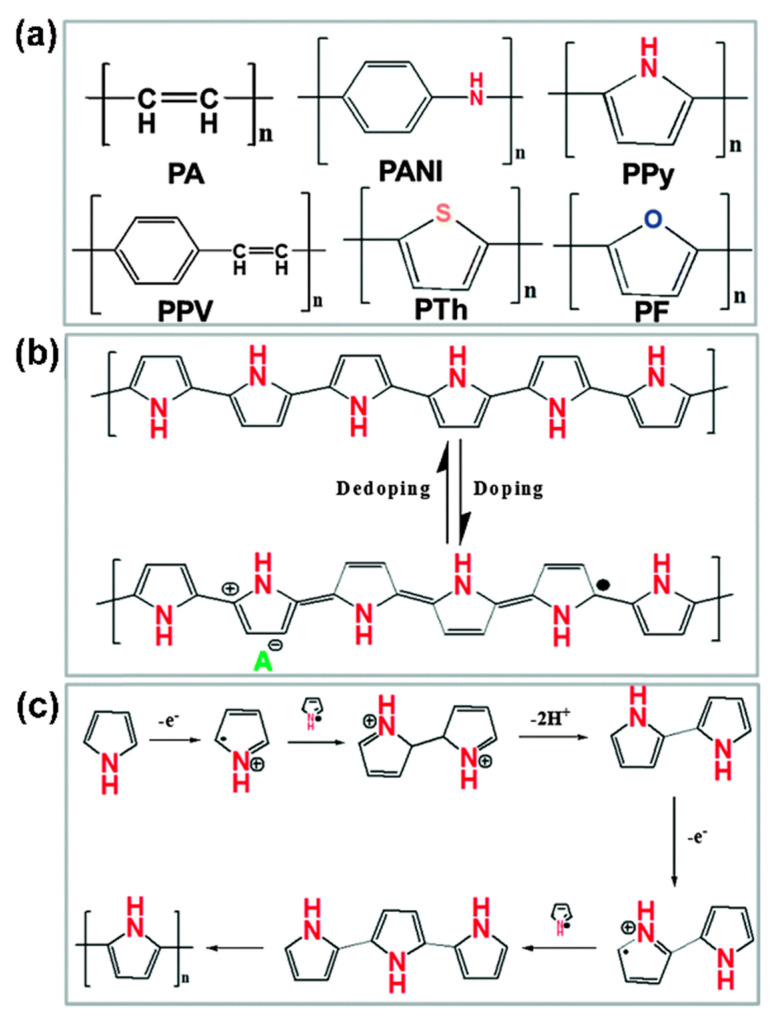
(**a**) Chemical structures of typical conductive polymers. (**b**) Doping and dedoping process of PPy. (**c**) Schematic of the polymerization mechanism of PPy [92].

Conventional commodity polymers are inherently insulating. The field of conductive polymers was revolutionized by the groundbreaking finding that halogen-doped polyacetylene ((-CH=CH-)_n_) demonstrates high electrical conductivity, which led to the Nobel Prize in Chemistry in the year 2000 [91]. The essential characteristic of conductive polymers is conjugation, which involves alternating single and double bonds along the polymer chain. Therefore, the synthesis of π-conjugated chains is fundamental for the science and technology of conductive polymers. As shown in Figure 4a [92], typical conductive polymers include polyacetylene (PA, 3–1000 S·cm^−1^), polyaniline (PANI, 0.01–5 S·cm^−1^), polypyrrole (PPy, 0.3–100 S·cm^−1^), polythiophene (PTh, 2–150 S·cm^−1^), poly (phenylenevinylene) (PPV, 10^−3^–100 S·cm^−1^), and others. Neural conjugated polymers typically have low conductivity, ranging from 10^−10^ to10^−5^ S·cm^−1^. However, this can be significantly increased, up to 10^4^ S·cm^−1^, through chemical or electrochemical redox reactions known as ‘‘doping’’. For example, the conductivity of PPy can be enhanced from ~0.1 S·cm^−1^ in its undoped state to 100 S·cm^−1^ after doping (Figure 4b). The proposed polymerization mechanism of PPy is shown in Figure 4c. The process starts with the oxidation of a monomer to form a radical cation, which then couples with another radical cation to form a dimer after losing two protons. The dimer undergoes further oxidation and couples with additional radical cations, leading to the formation of oligomers. This sequence of oxidation, coupling, and deprotonation proceeds until the final polymer is obtained [9].

**Figure 5:**
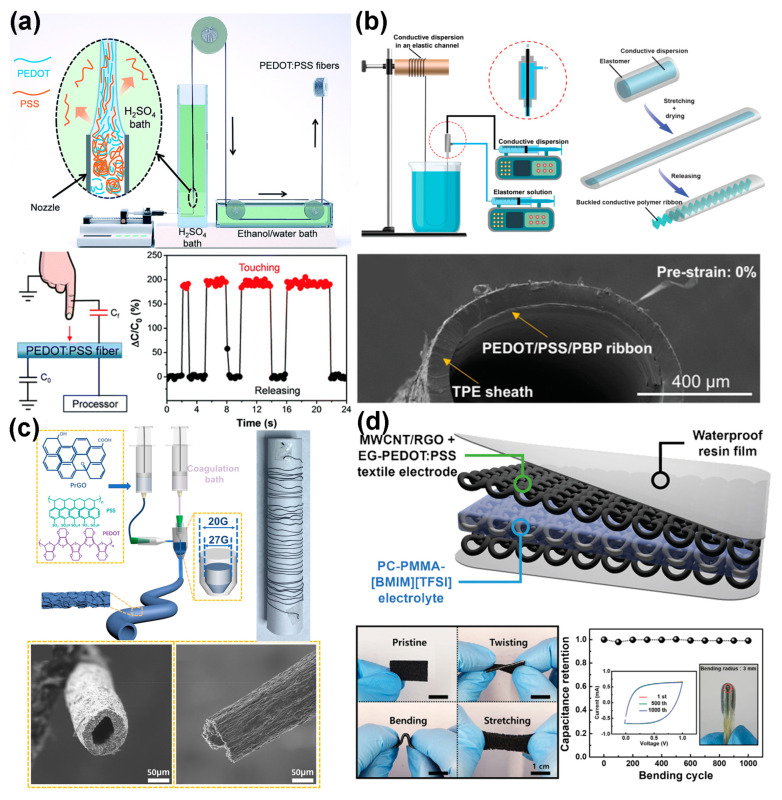
(**a**) Schematic of the modified setup for wet-spinning PEDOT:PSS fibers and their application [93]. (**b**) Coaxial wet-spinning process to encapsulate conductive dispersion in an elastic TPE channel, with an SEM image of the coaxial fiber [94]. (**c**) Schematic of the HCFs fabrication process, including the cross-sectional and side-view SEM images of HCFs-50 [18]. (**d**) Schematic of the stretchable spandex/nylon fabric coated with the highly conductive polymer PEDOT:PSS [95].

Conductive polymers are a novel class of organic functional materials that combine the desirable properties of conventional polymers with those of metals or semiconductors. Their tunable physical and chemical properties, excellent biocompatibility, and ease of fabrication make them promising candidates for various fundamental and applied fields [19,44,58,85]. As shown in Figure 5a, Zhang et al. [93] proposed a simple one-step method to prepare highly conductive poly (3,4-ethylenedioxythiophene):poly (4-styrenesulfonate) (PEDOT:PSS) fibers. These fibers, with a diameter of approximately 15 μm, achieved an electrical conductivity of 3828 S·cm^−1^, comparable to the thinnest PEDOT:PSS film to date (4380 S·cm^−1^ for a 100 nm film). Moreover, the dry fibers exhibited an outstanding tensile strength of 434.8 MPa and a breaking strain of approximately 25.4%. They functioned as capacitive touch sensors, showing a 200% increase in capacitance within 20 milliseconds upon contact with a finger. The tensile potential of conductive polymers is challenging to fully realize through traditional fiber drawing processes, but significant improvements can be made through processing modifications and optimized fiber structure design. For example, as shown in Figure 5b, Zhou et al. [94] employed a coaxial wet-spinning assembly method to continuously spin coaxial fibers consisting of a thermoplastic elastomer outer layer and a core of PEDOT/PSS/PBP aqueous solution. These fibers exhibited reversible stretchability up to 680% with less than 4% change in resistance. A “solution stretching-drying-buckling” approach was then applied to achieve the desired morphology of a conductive layer with a high strain capacity ($\varepsilon_{p}$ = 700%). The resistance of the fibers remained almost unchanged over 1766 cycles. Furthermore, He et al. [18] developed hollow composite fibers (HCFs: length of 1.1 m) composed of a mixture of partially reduced graphene oxide (PrGO) and PEDOT:PSS via coaxial wet-spinning. The HCFs-50-based fiber-shaped supercapacitors (FSCs) exhibited high specific energy density and stable cycling performance over 10,000 cycles. The fiber-shaped dye-sensitized solar cells (FDSSCs) based on HCFs-50 demonstrated reliable photovoltaic performance, which can stably power multiple commercial electronics after photo-charging. This offers a promising approach for developing flexible self-powered systems (Figure 5c). Conductive polymer fibers are considered as promising candidates for stress sensors due to their excellent flexibility. As shown in Figure 5d, Lee et al. [95] fabricated a high-performance textile-based stretchable supercapacitor that remained stable across a wide temperature range from −30 to 80 °C. The supercapacitor used a stretchable spandex/nylon fabric coated with the highly conductive polymer PEDOT:PSS as a current collector, enabling it to endure repeated stretching and bending. Integrated into a nylon glove, the supercapacitor powered a micro-light-emitting diode that operated stably regardless of finger bending.

Conductive polymers are appealing as conductive fillers due to their corrosion resistance and their electrical, mechanical, and optical properties, which complement conventional conductive materials. However, several challenges remain: I. No technique currently achieves both high conductivity and stretchability in PEDOT without small molecule additives. II. Non-hydrogel-based PEDOT needs a larger elastic range for reversible stretchability. III. The effects of humidity, temperature, stretching rate, and morphology on the mechanical and electronic properties of new stretchable materials need further investigation [19].

### 1.5. Ionic Conductor-Based SCCFs

Although electronic conductive fillers are commonly used for fabricating stretchable conductive fibers, some challenges remain, including achieving interface compatibility between fillers and matrices, balancing mechanical performance with electrical properties, and managing the leakage risk of conductive materials [7]. Ionic conductors, which are innovative interdisciplinary materials integrating bioscience and electrochemistry, functioning by facilitating the directional migrations of free ions driven by voltage differences. Their conductivity depends on the concentration of efficient free ions within the conductive pathways [96]. Deformation has minimal impact on ionic conduction, allowing ionic conductors to surpass the limitation of percolation theory. Additionally, ionic conductors are biocompatible and can mitigate severe side effects caused by voltage drops in biological tissues. As a result, various ionic conductors have been utilized in bionic skins, stretchable energy devices, and artificial muscles [3,14,64,97].

**Figure 6:**
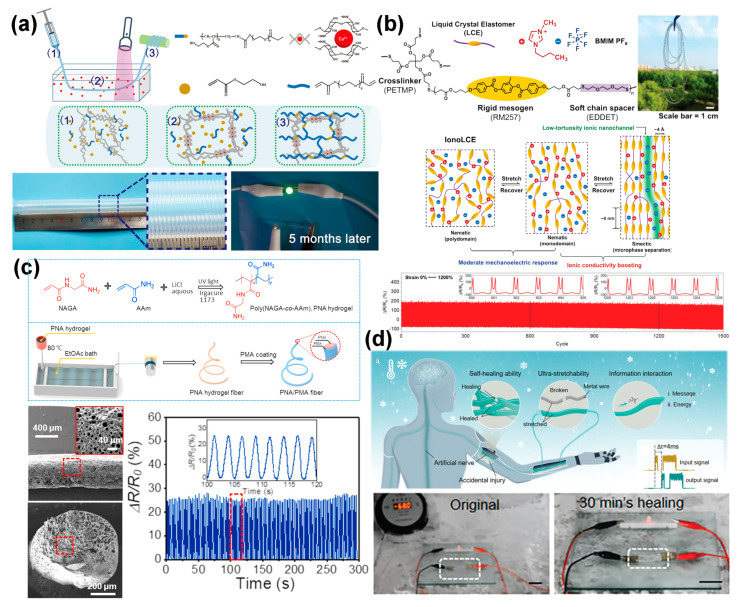
(**a**) Schematic of the molecular design and evolution of organohydrogel fibers, with photographs of a long single fiber collected onto a continuously winding drum spool and an LED powered directly by this fiber [98]. (**b**) Molecular structures of IonoLCE, composed of ionic liquid and LCE network, and the working mechanism for ion transport modulation, along with real-time resistance changes of this fiber under 1500 cycles [99]. (**c**) Preparation of PNA/PMA core–sheath fibers, including SEM images of the fiber and its cycling stability as a strain sensor [23]. (**d**) Ionic fibers with frost resistance for applications in artificial nerve, demonstrating electrical self-healing capacity at −68 °C [29].

SCCFs are essential components for the development of stretchable electronics. In comparison to electron-conductive fibers, ion-conductive hydrogel fibers display superior characteristics such as greater stretchability, transparency, and minimal resistance variation under strain. As shown in Figure 6a, Song et al. [98] designed a novel continuous wet-spinning process to fabricate organohydrogel fibers (0.765 S·m^−1^) that are stretchable, transparent, and conductive. These hydrogel fibers exhibited superior mechanical properties (400 ± 9.6% strain at break) and high dynamic mechanical stability. They were used to fabricate strain sensors that accurately capture high-frequency (4 Hz) and high-speed (24 cm·s^−1^) motions with minimal drift over 1000 stretch–release cycles, making them effective for detecting fast cyclic motions such as engine valves, which previous conductive fibers struggled with. However, the organohydrogel fibers have poor tensile stress and hardness, limiting their engineering applications. Yao et al. [99] reported that ionotronic liquid crystal elastomer (IonoLCE) fibers, produced by introducing ionic liquid (IL) into the LCE network, form low-tortuosity ion transport nanochannels through an alignment-induced microphase separation during stretching. This results in a dramatic enhancement in strain-induced ionic conductivity (1000-fold increase at 2000% strain). The IonoLCE fibers exhibited remarkable robustness, with high stretchability (up to ~2700% strain), outstanding elastic recovery (99% recovery from 1200% strain), and ultrahigh toughness (56.9 MJ·m^−3^). These fibers serve as durable strain sensors, distinguishing waveforms based on changes in output resistance (Figure 6b). The integration of functional polymers and ionic liquids not only improves the mechanical properties of the fibers but also enhances their functional characteristics. As shown in Figure 6c, Shuai et al. [29] fabricated conductive, stretchable, and self-healing fibers from acrylamide (AAm) and N-acryloylglycinamide (NAGA) (poly(NAGA-co-AAm), PNA) using a continuous dry–wet spinning approach. The PNA hydrogel fibers exhibited strong tensile strength (2.27 MPa), stretchability (900%), high conductivity (0.69 S·m^−1^), and self-healing capabilities. PNA/PMA core–shell fibers, created by coating PNA fibers with elastic poly (methyl acrylate) (PMA), showed high resistance to moisture evaporation and absorption. These PNA/PMA fibers were used to construct a triboelectric nanogenerator (TENG) textile that could convert mechanical motion into electric power. The stretchable TENG textile showed its potential as a power source for wearable electronics. To enhance the environment adaptability of ionic conductor fibers, Wang et al. [30] proposed a simple, general method to improve hydrogels. This ionic hydrogel showed outstanding conductivity and frost resistance, maintaining stable performance even under −80 °C. Artificial nerve fibers mimicking the structure and function of myelinated axons were developed, enabling real-time, high-fidelity information transmission through potential-gated mechanisms. These artificial nerve fibers exhibited remarkable potential for soft robotic applications due to their deformability and self-healing capacities to cope with diverse and harsh environments (Figure 6d).

To sum up, ionic stretchable conductive fibers represent a novel class of materials with significant advantages, including skin-like properties, high flexibility, mechanical robustness, and biocompatibility. These characteristics make them well-suited for advanced wearable devices and electronic skins. However, the limited spinnability of ionogels and their precursors obstructs the continuous production of fibrous ionogels, as the network formation of ionogels takes significantly longer than the fiber formation process. Moreover, using very small ions as charge carriers in ionic conductors leads to a low gauge factor. To address these challenges, future research should focus on developing more stable ionic conductors or exploring smart-encapsulation techniques to improve their environmental stability [3,7,30,99].

## 2. Challenges and Prospects

In summary, we have systematically reviewed the conductive mechanisms, materials selection, fabrication strategies, and different applications of SCCFs. Compared to the traditional framework of electronic devices, SCCFs offer significant advantages in conductivity, tunability, breathability, and mechanical compliance, which are crucial for maintaining stable functionality in wearable devices and electronic skin. Given their primary application scenarios, such as skin contact, clothing integration, and attachment to bio-interfaces, the future development of SCCFs should focus on materials with multifunctionality, biosafety, and compatibility.

Furthermore, integrating diverse devices onto SCCF substrates to create multifunctional systems presents key challenges, including accurate integration and efficient production. Additionally, interface conformity, all-in-one integration, and seamless adaptation need to be considered. For device manufacturing, the primary challenges are miniaturization, precise fabrication, and scalable production. To address these issues, the following solutions could be effective:

(I) Interface adaptability between devices and SCCFs-based substrates: repeated operation and long-term use can cause delamination between functional layers, causing performance deterioration, signal distortion, or potential safety concerns. Enhancing the interface adaptability of electrode materials to SCCFs-based substrates is crucial. Strategies such as matching Young’s modulus, modifying wettability, and enhancing interface adhesion are vital. Therefore, exploring printable conductive materials with self-attachability, self-healing capabilities, and dynamic electrical stability is a promising direction.
(II) Balancing conductivity and stretchability of SCCFs: in addition to enhancing interface compatibility, the stability between electrical conductivity and stretchability in SCCFs is also crucial. To stabilize conductive components in SCCFs during post-treatment, strategies such as appropriate binder adhesion or cosolvent etching could be effective. For direct spinning of SCCFs, introducing ionic liquid conductive fillers to dynamically compensate the conducting network could enhance conductive stability during deformation.
(III) Precise adjustment and large-scale production: emerging spinning and 3D-printing technologies complement traditional processes by enhancing device structure and functionality. These innovations hold particular promise for the miniaturization and precise manufacturing of devices. Moreover, enhancing the mechanical strength and processability of novel SCCF materials to align with established textile-engineering techniques would significantly advance the scalable production of related devices.
